# Supplementary material for: A squarate-pillared titanium oxide quantum sieve towards practical hydrogen isotope separation
Source: Nat Commun. 2023 Jul 13;14:4189. doi: 10.1038/s41467-023-39871-x (PMC10344961; doi:10.1038/s41467-023-39871-x)
Supplement: Supplementary file 1 — Supplementary Information [file 41467_2023_39871_MOESM1_ESM.pdf]

Supplementary Information

A Squarate-pillared titanium oxide quantum sieve towards practical hydrogen isotope separation

Qingqing Yan et al

Supplementary Figures

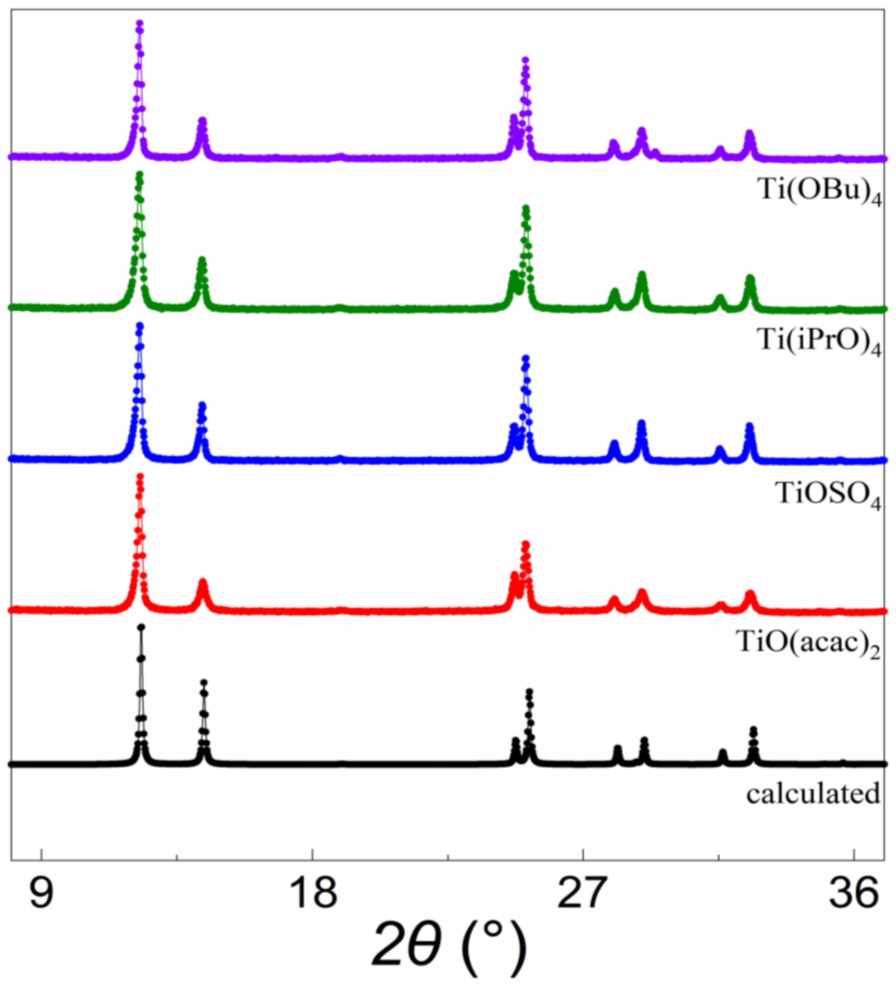

Supplementary Figure 1 | PXRD patterns of USTC-700 samples synthesized with different titanium precursors under the same reaction condition.

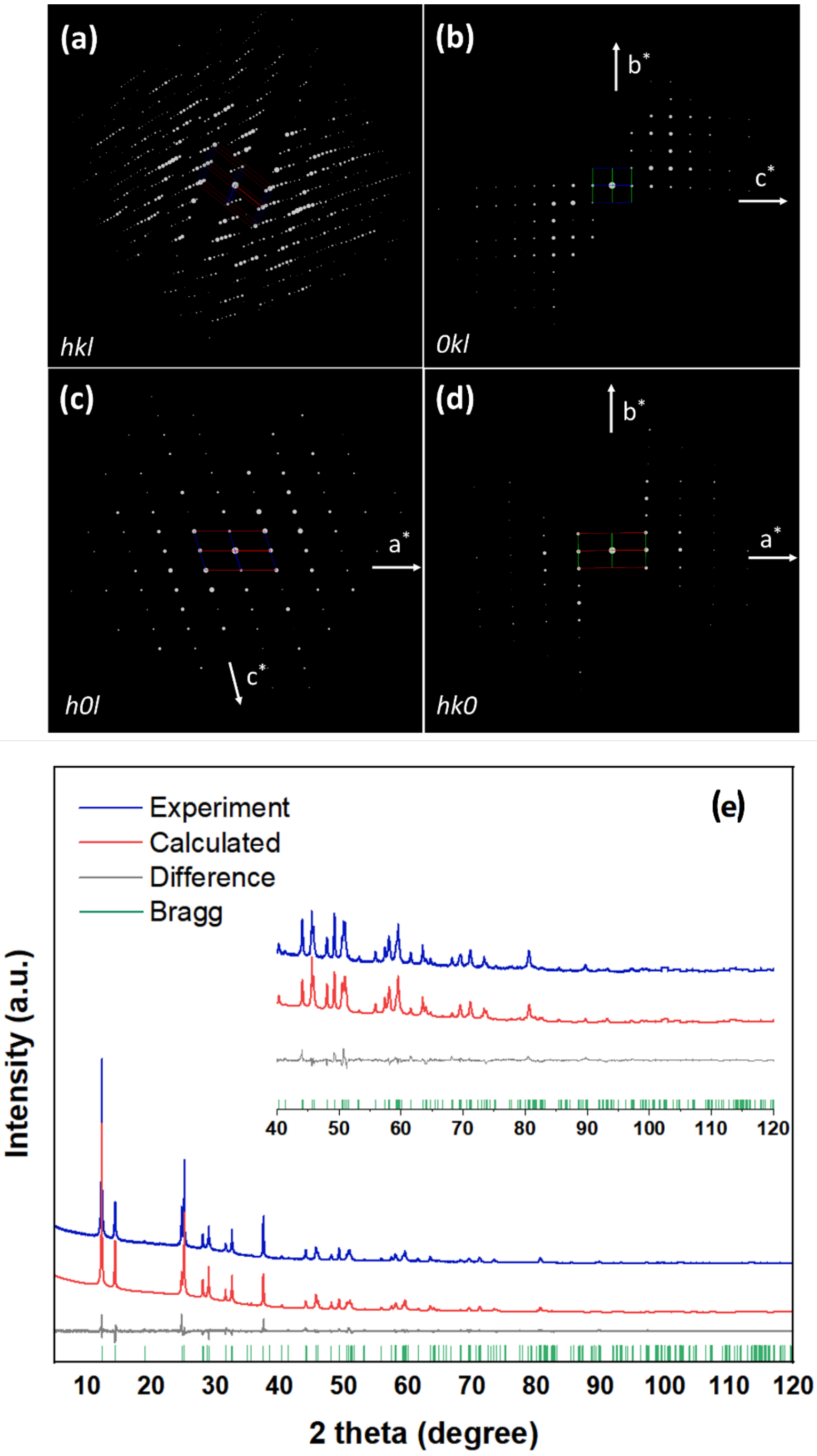

Supplementary Figure 2 | Crystal structure determination of USTC-700. (**a**) Reconstructed 3D reciprocal lattice of USTC-700 from the cRED data. (**b**)–(**d**) Three two-dimensional slices 0*kl*, *h*0*l*, and *hk*0 extracted from the reconstructed reciprocal lattice. (**e**) Final Rietveld refinement plots of USTC-700. The experiment, calculated, and difference curves are in blue, red and gray, respectively. The vertical bars indicate the positions of Bragg peaks (Cu Kα_1, λ = 1.5406 Å). The inset shows the high angle part of the profiles.

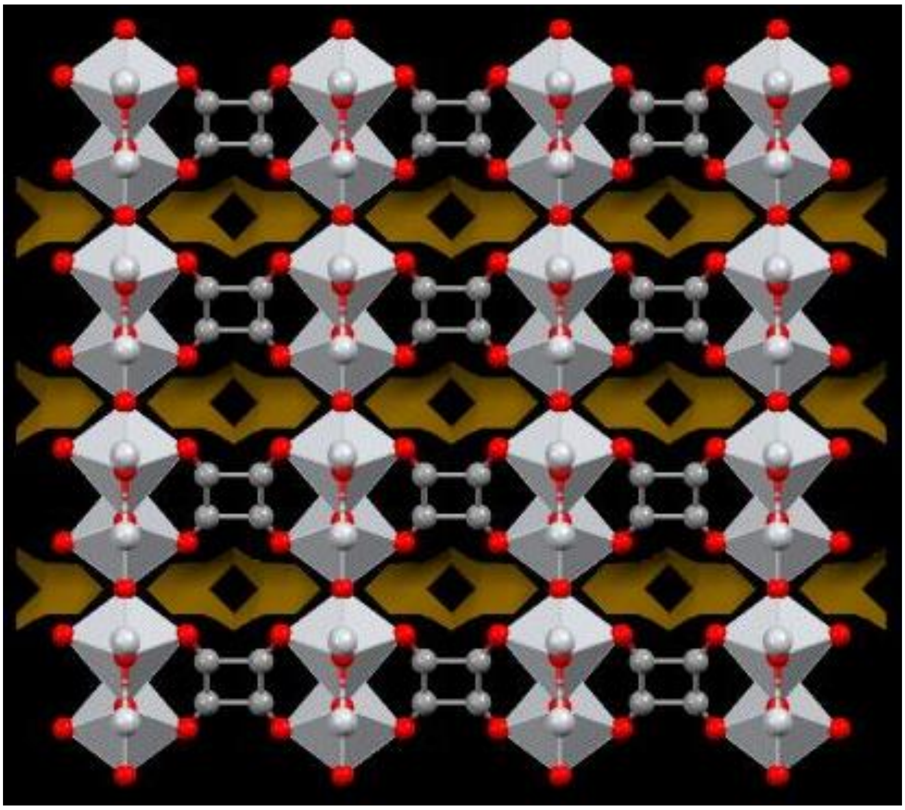

Supplementary Figure 3 | Square micropores running along the *a*-axis of the USTC-700 structure (Titanium in gray polyhedron; carbon atoms in gray; oxygen atoms in red; and the accessible cavities are represented in gold).

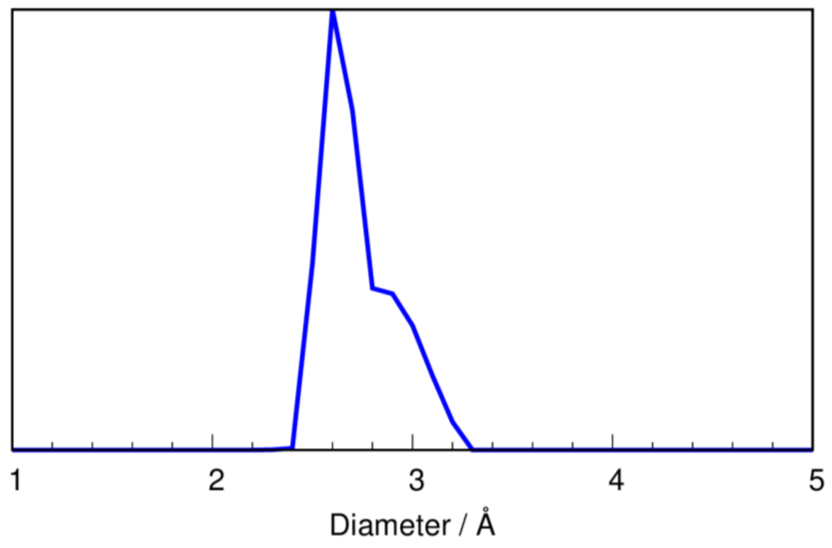

Supplementary Figure 4 | Pore size distribution of the USTC-700 framework calculated for the DFT optimized geometry.

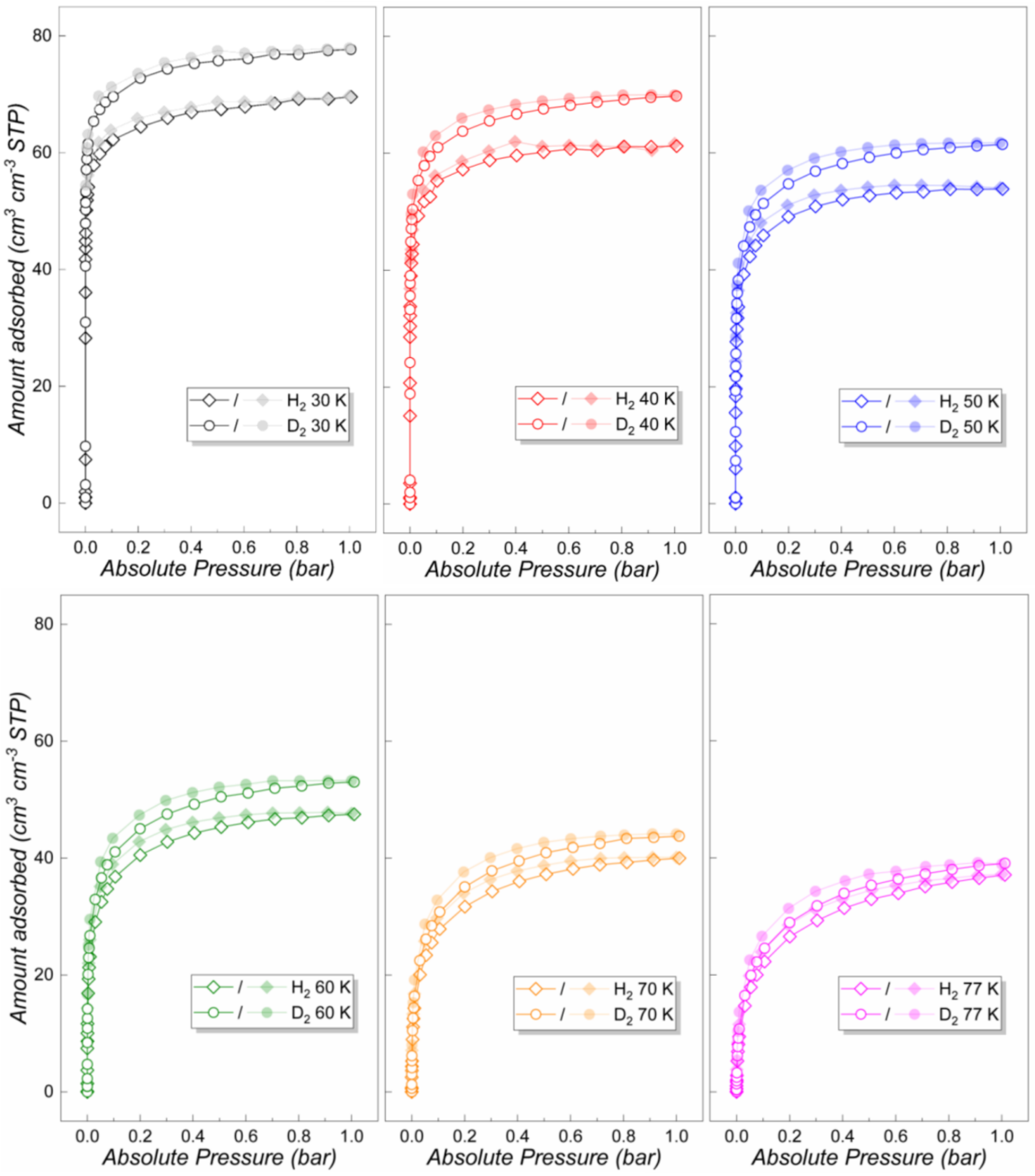

Supplementary Figure 5 | H_2 and D_2 single component sorption isotherms collected at 30 K, 40 K, 50 K, 60 K, 70 K and 77 K, respectively.

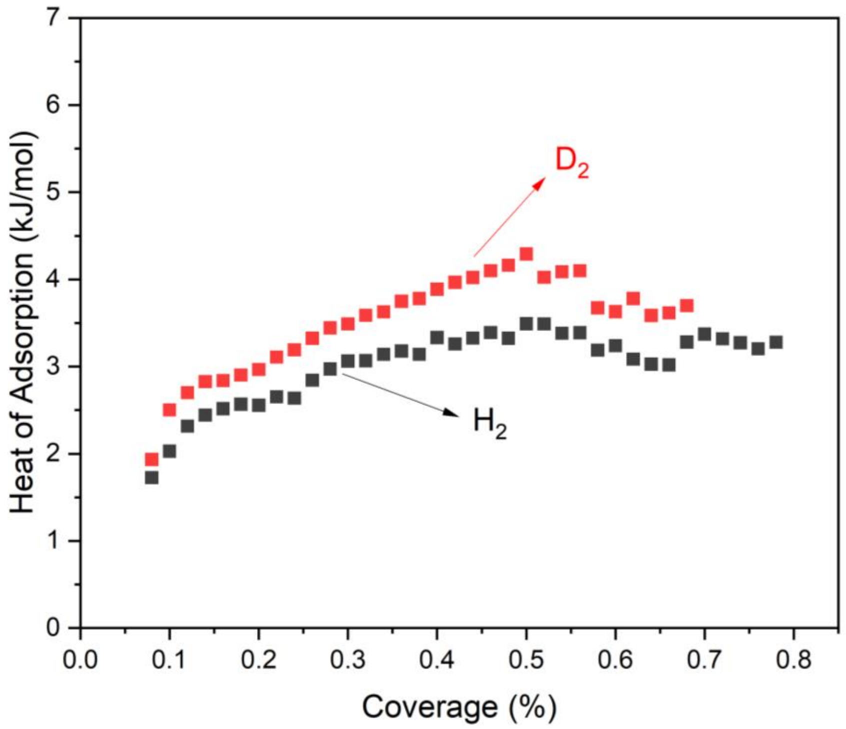

Supplementary Figure 6 | Isosteric heat of H_2 and D_2 adsorption on USTC-700 as function of coverage.

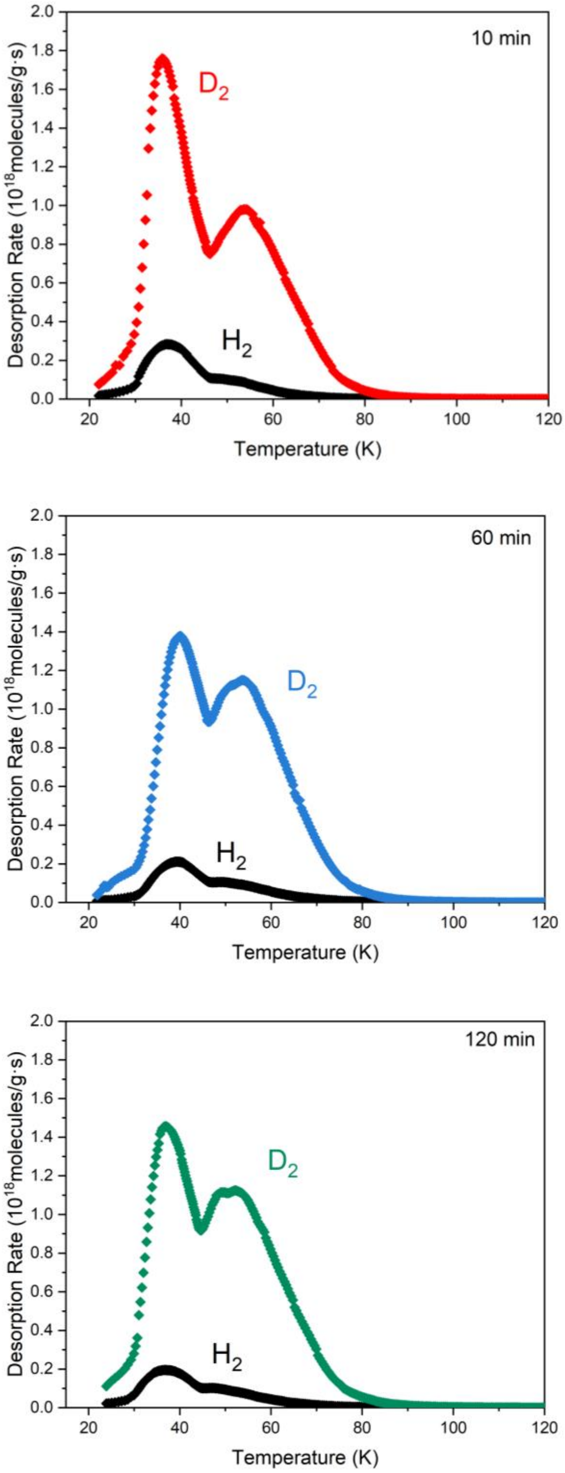

Supplementary Figure 7 | Thermal desorption spectra (TDS) of USTC-700 obtained after exposure to a 10 mbar 1:1 D_2/H_2 isotope mixture at 30 K for an exposure time from 10 to 120 min. The deuterium (red) and hydrogen (black) desorption spectra after evacuation for different exposure time were measured for a heating rate of 0.1 K/s.

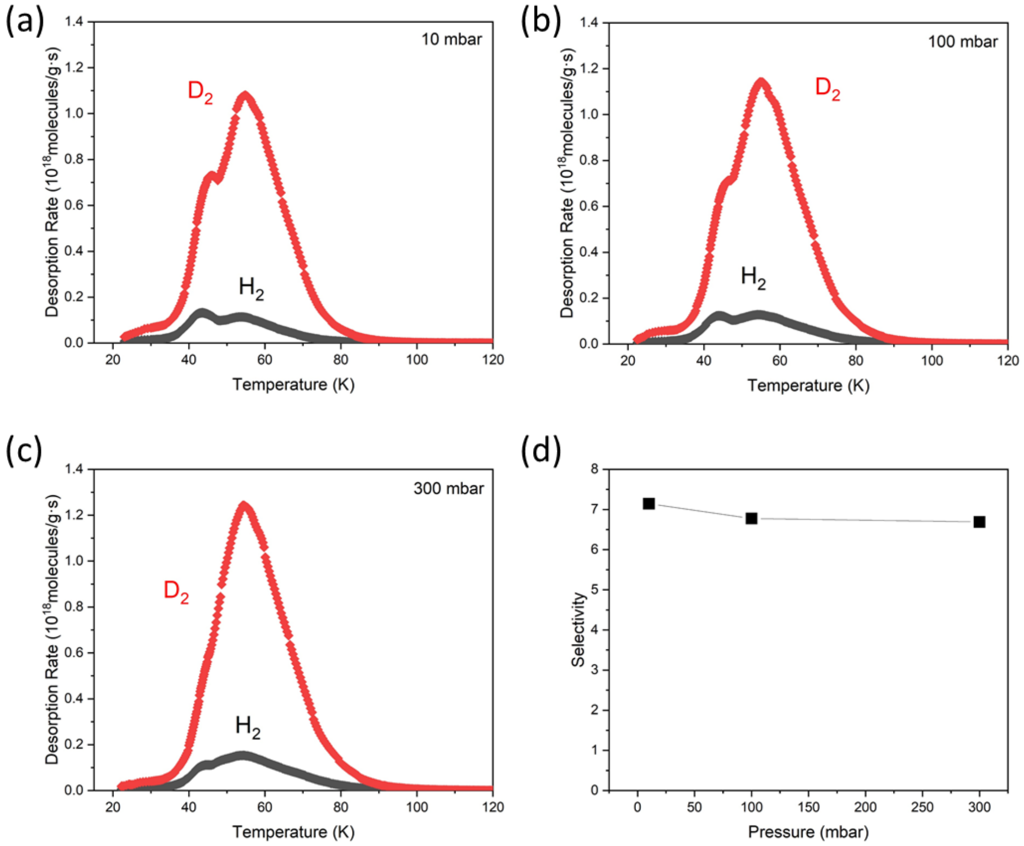

Supplementary Figure 8 | Thermal desorption spectra (TDS) of USTC-700 obtained after exposure to a 1:1 D_2/H_2 isotope mixture at 40 K for an exposure time of 10 min. (**a**-**c**) The deuterium (red) and hydrogen (black) desorption spectra after evacuation of different exposure pressure (10, 100, and 300 mbar) were measured for a heating rate of 0.1 K/s. (**d**) D_2/H_2 selectivity as a function of exposure pressure.

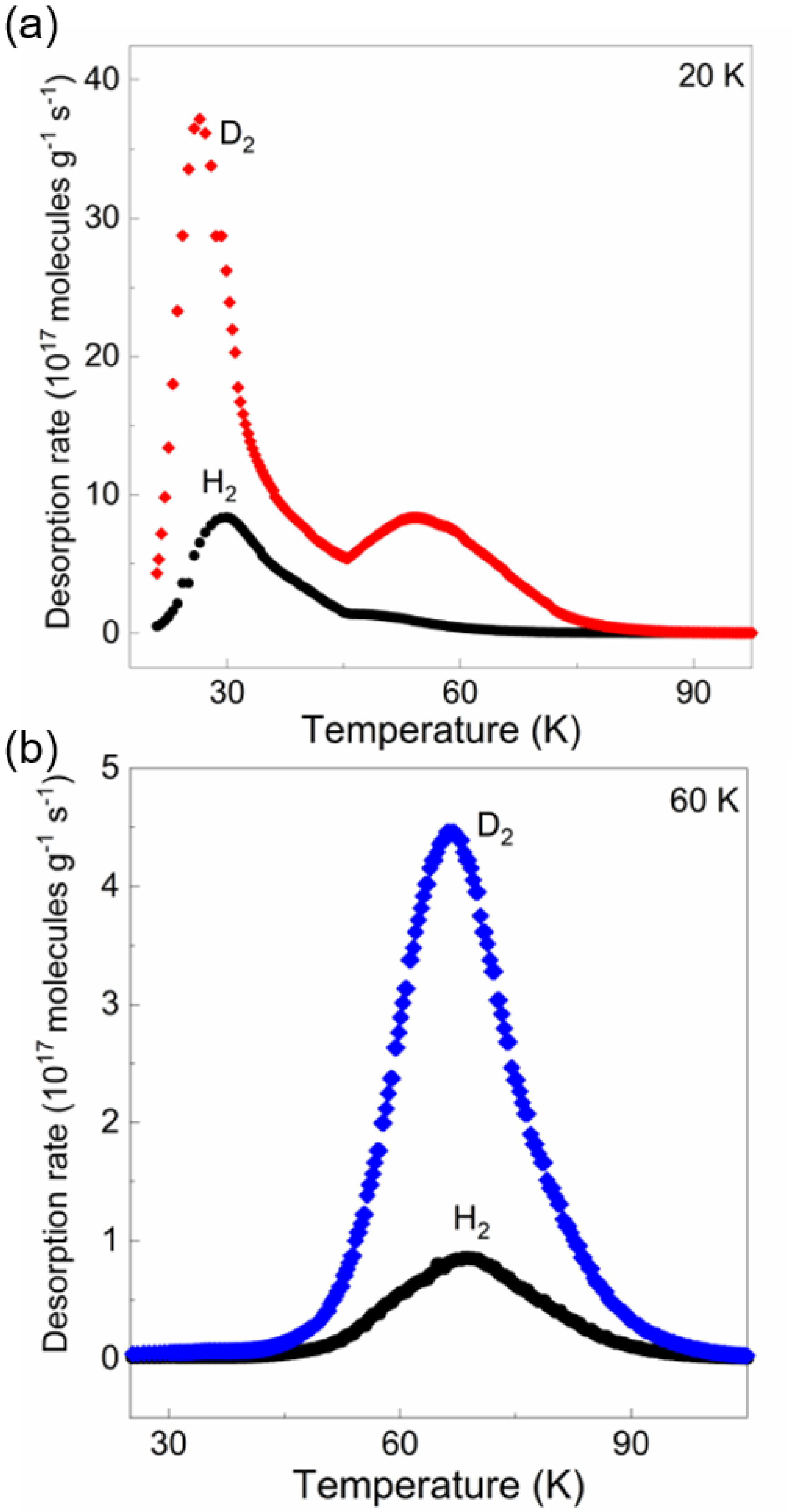

Supplementary Figure 9 | Cryogenic thermal desorption spectroscopy (TDS) results of USTC-700 obtained after the exposure to a 10 mbar 1:1 mixture of H_2 and D_2 for 10 minutes at 20 K (**a**) and 60 K (**b**).

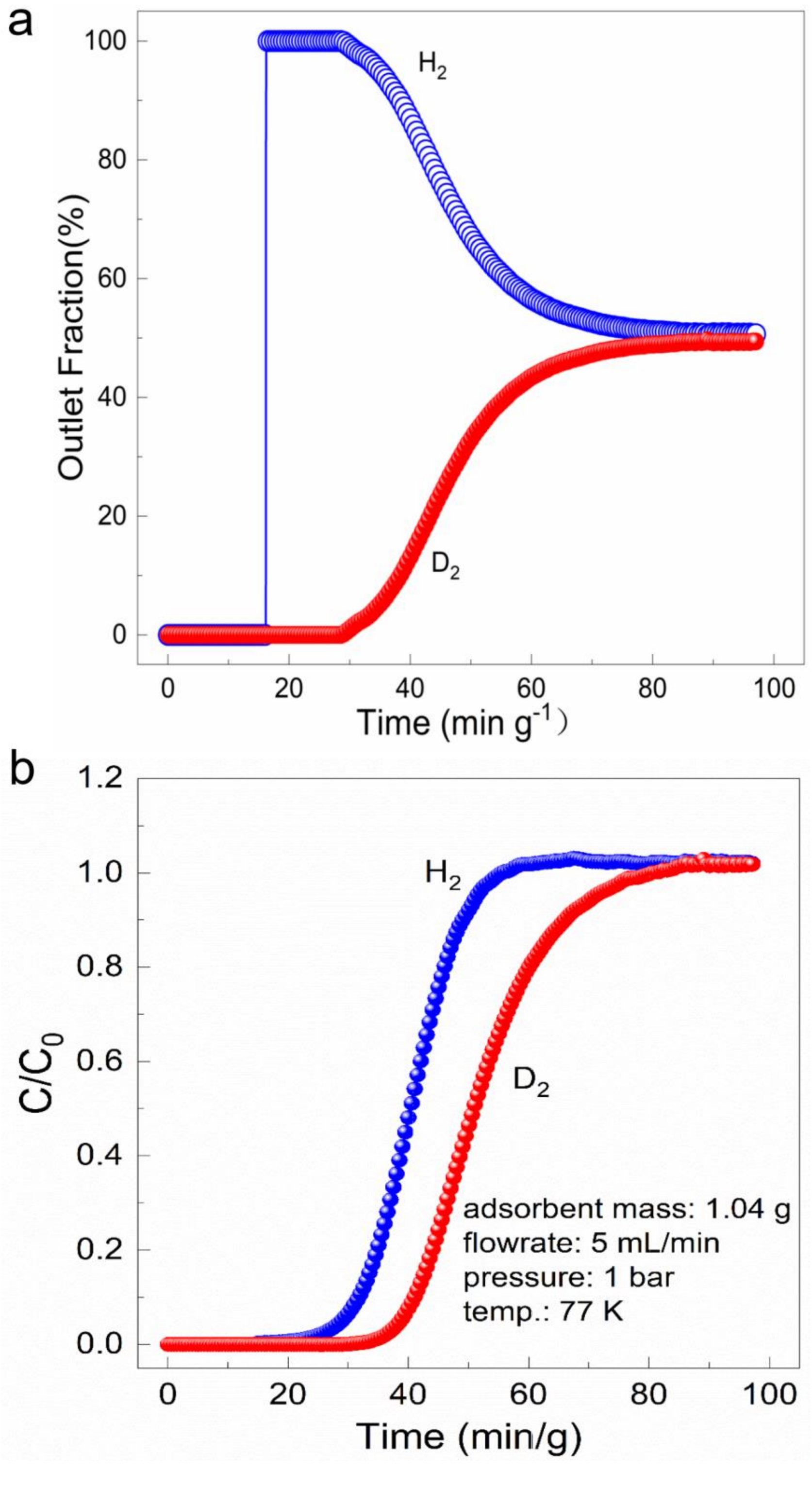

Supplementary Figure 10 | (**a**) The dynamic breakthrough curve and (**b**) Column breakthrough curves of USTC-700 at 77 K for the mixed gases of $H_2/D_2/Ne$ (1/1/98 vol.%).

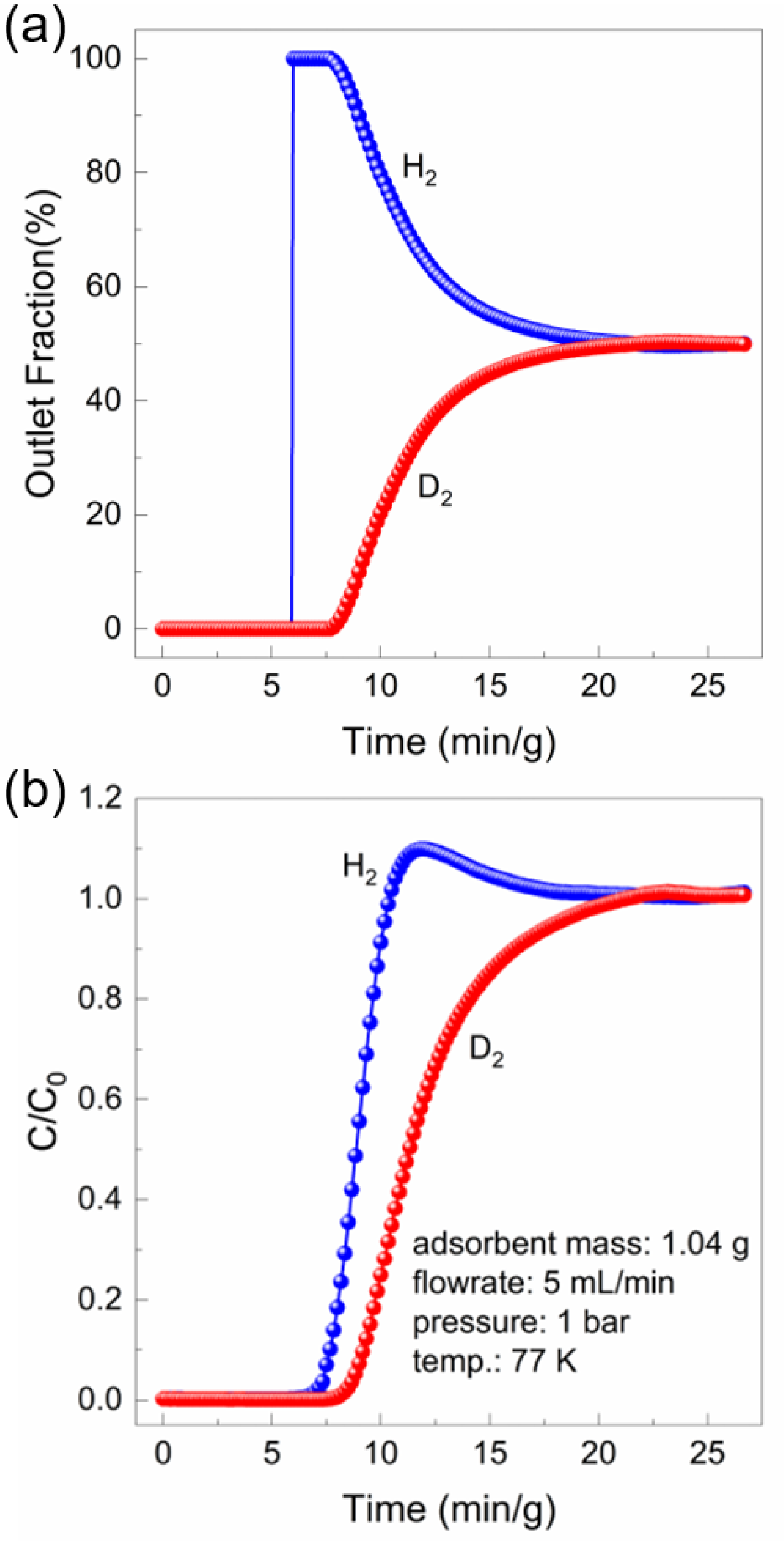

Supplementary Figure 11 | (**a**) The dynamic breakthrough curve and (**b**) Column breakthrough curves of USTC-700 at 77 K for the mixed gases of H_2/D_2/Ne (10/10/80 vol.%).

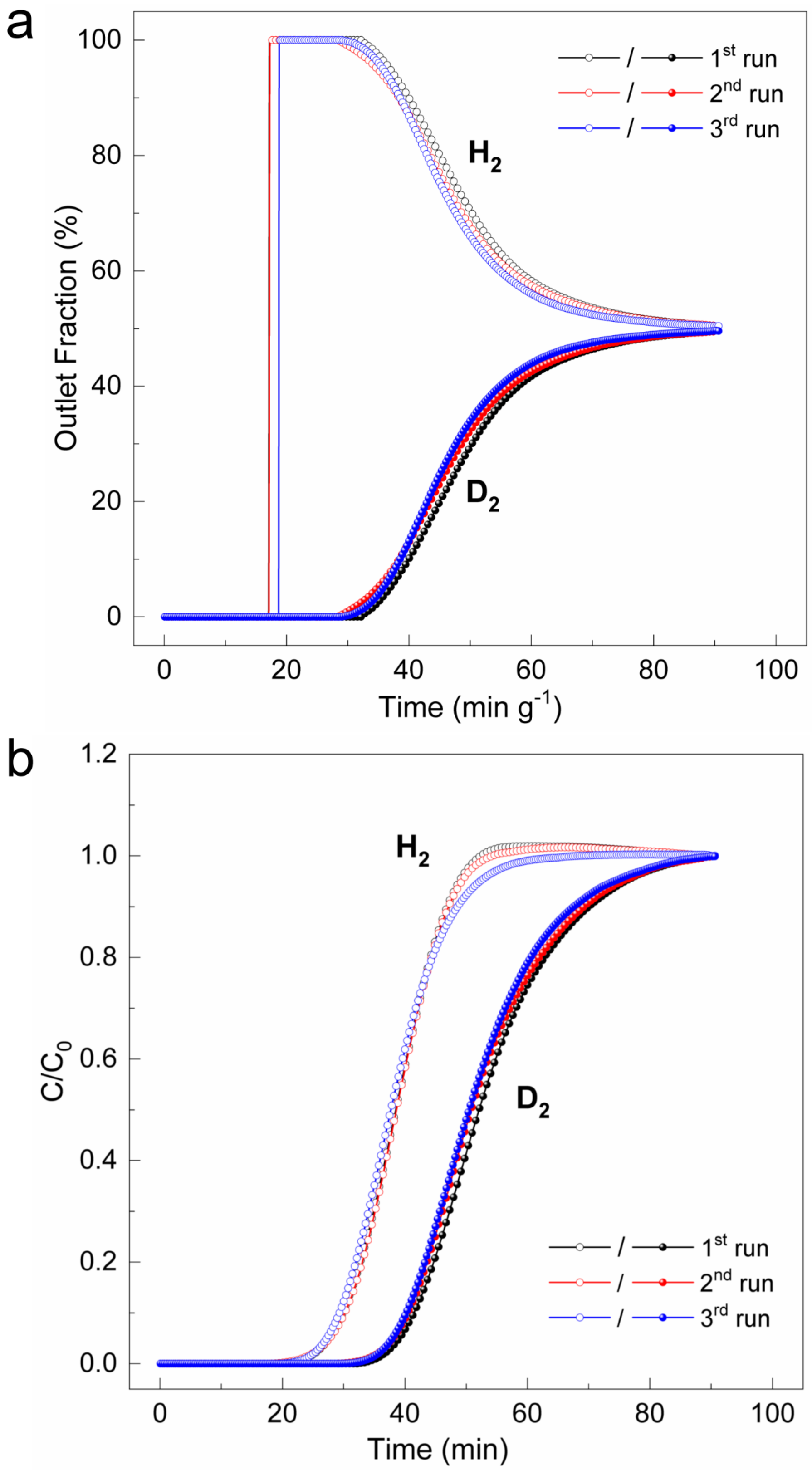

Supplementary Figure 12 | (**a**) The cycling dynamic breakthrough curves and (**b**) cycling column breakthrough curves of USTC-700 at 77 K for the mixed gases of $H_2/D_2/Ne$ (1/1/98 vol.%).

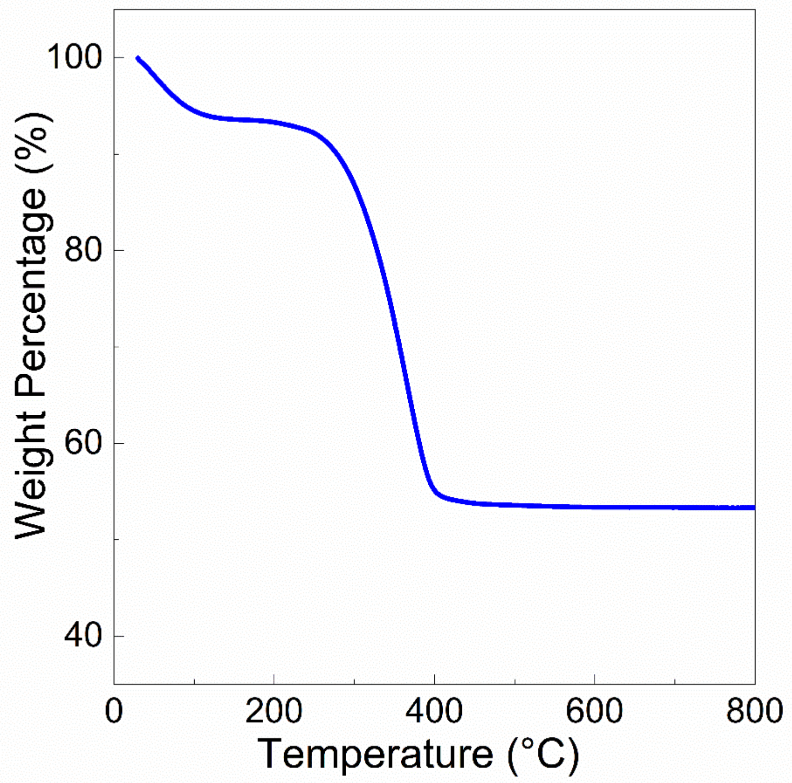

Supplementary Figure 13 | TGA curve of USTC-700.

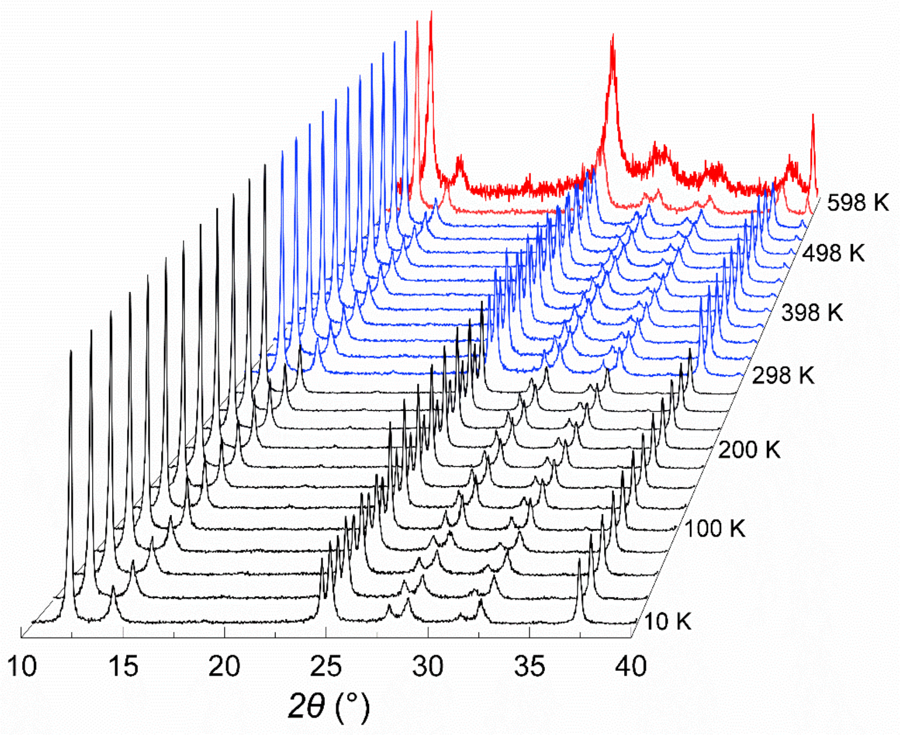

Supplementary Figure 14 | Temperature-dependent PXRD patterns of USTC-700 showing that the long-range order of the structure can be stable up to around 575 K.

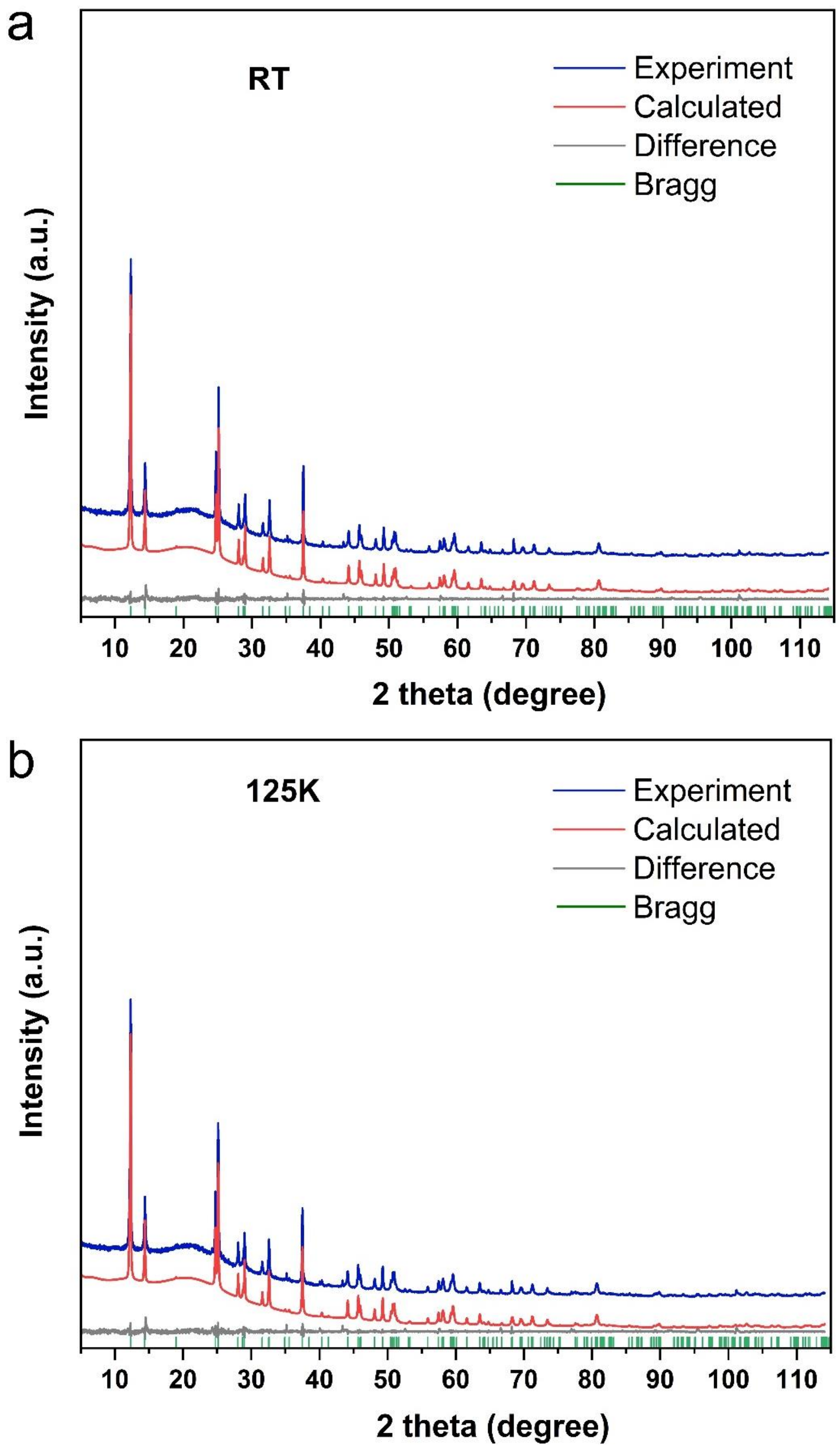

Supplementary Figure 15 | Indexing and pattern fitting of the PXRD data collected on the sample sealed in capillary at room temperature (**a**) and 125 K (**b**), respectively.

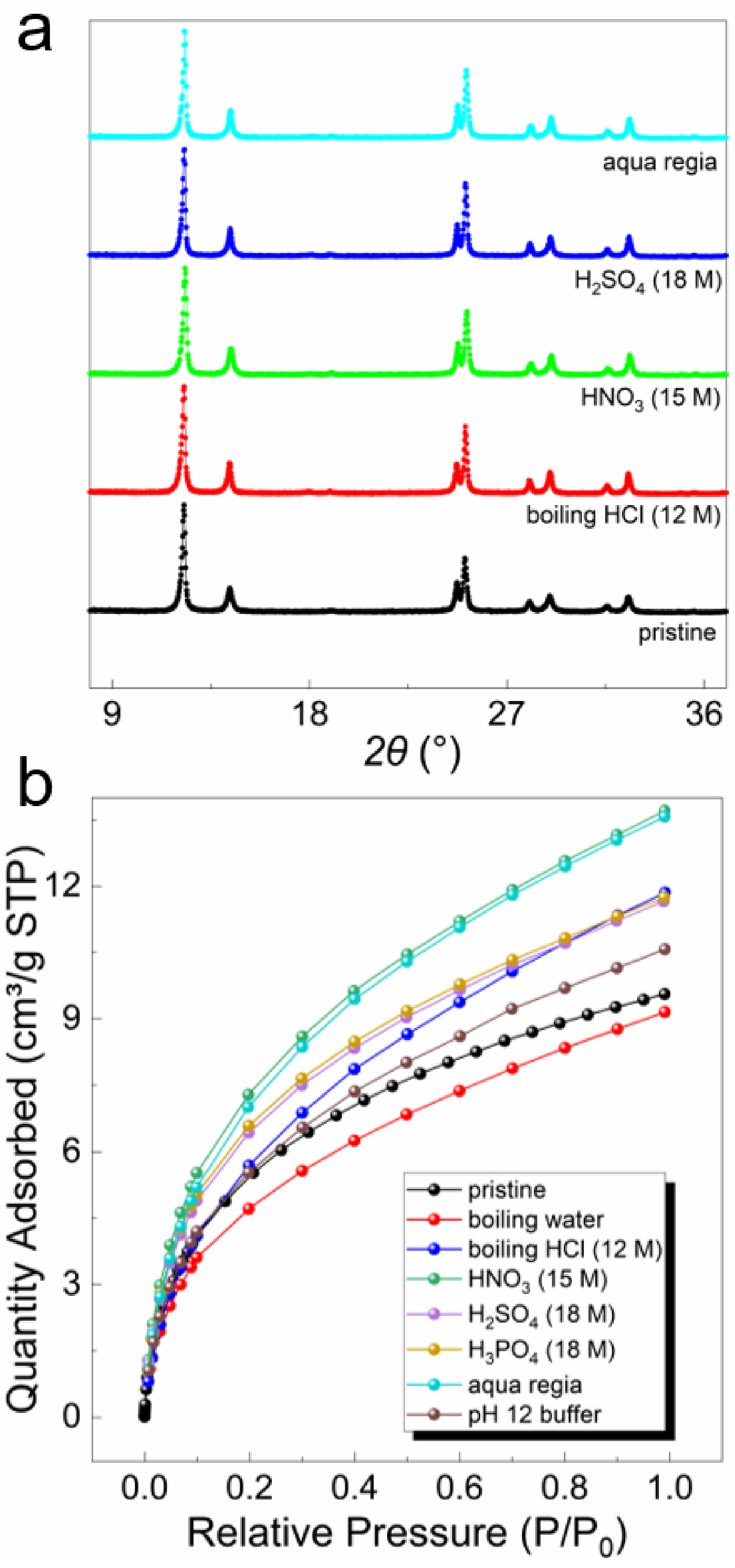

Supplementary Figure 16 | (**a**) PXRD patterns and (**b**) CO_2 adsorption isotherms of USTC-700 samples before and after chemical stability test under harsh conditions.

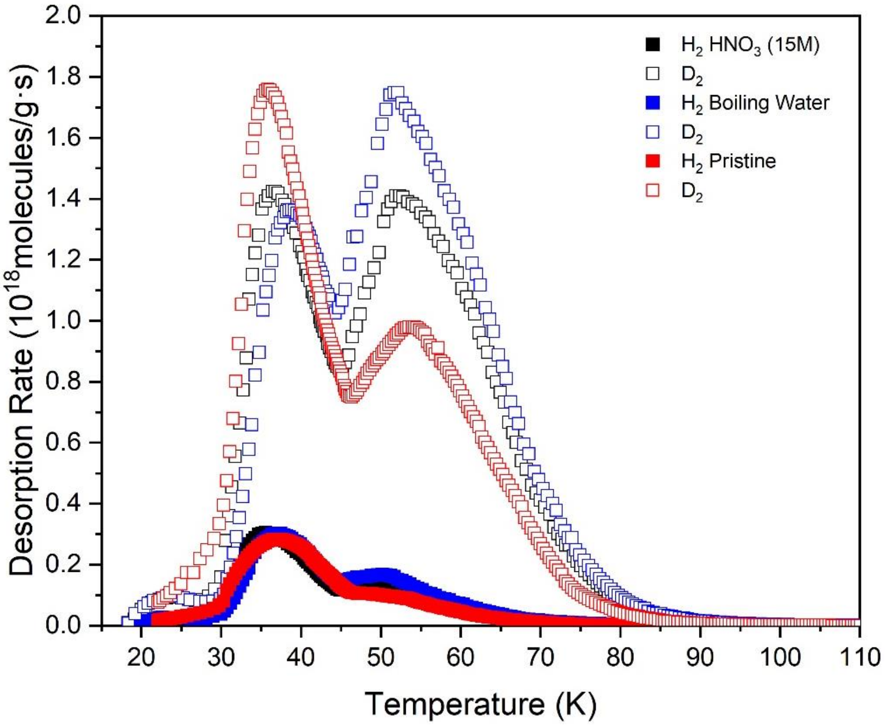

Supplementary Figure 17 | TDS results of USTC-700 samples after chemical treatments (please note that the intense of each peak varies, since each batch of sample are not 100% homogenous, and TDS is extremely sensitive to minor variation).

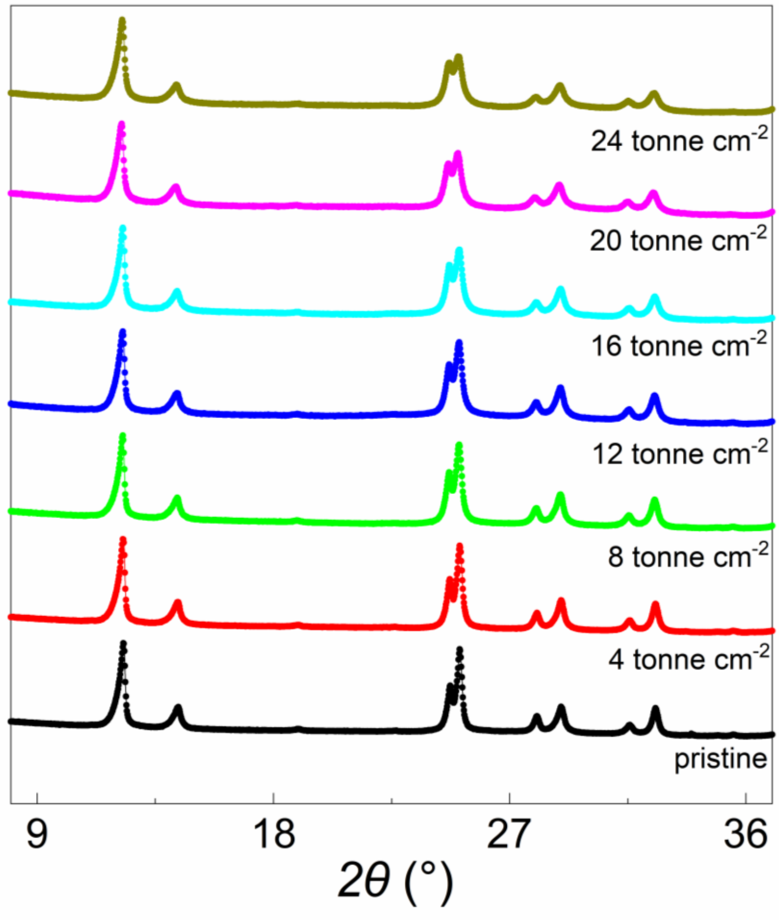

Supplementary Figure 18 | PXRD patterns of USTC-700 samples before and after mechanical stability test.

Supplementary Tables

Supplementary Table 1 | Crystallographic details of cRED dataset for the structure solution of USTC-700.

**Sample**	USTC-700
**Tilt range (°)**	-53.4 ~ 64.3
**Exposure time/frame (s)**	0.2
**Number of frames**	742
**Program for structure determination**	SHELXT
**Crystal system**	monoclinic
**Space group**	*P*2/*m*
a **(Å)**	3.69
b **(Å)**	7.21
c **(Å)**	6.41
α **(°)**	90.30
β **(°)**	106.36
γ **(°)**	90.54
V **($Å^3$)**	163.9
**Resolution (Å)**	0.9
**Completeness (%)**	85.1
**R_{int} (%)**	16.51
**Unique Reflections**	217
**Parameters**	14
**Restraints**	0
**R_1 (Fo > 4 sig (Fo))**	26.87 %
**GOF**	2.18

Supplementary Table 2 | Atomic coordinates of USTC-700 determined by cRED.

	**x**	**y**	**z**
**Ti1**	0.354608	0.500000	0.211460
**O1**	0.824150	0.500000	0.135350
**O2**	0.500000	0.500000	0.500000
**O3**	0.362010	0.219248	0.224596
**C1**	0.222161	0.122703	0.079596

Supplementary Table 3 | Crystallographic details of Rietveld refinements of USTC-700.

**Sample**	USTC-700
**Space group**	*P*2/*m*
a **(Å)**	3.6974 (2)
b **(Å)**	7.1931 (5)
c **(Å)**	6.4272 (5)
α **(°)**	90
β **(°)**	106.633 (4)
γ **(°)**	90
V **($Å^3$)**	163.78 (2)
R_p	2.59 %
R_{wp}	4.26 %
R_{bragg}	3.31 %
**GOF**	2.11

Supplementary Table 4 | Summary of hydrogen isotope selectivity and deuterium uptakes for various porous materials via KQS. The best performance of each sorbent was selected.

Compound	Deuterium uptake (mmol g^{-1})	Sorbent Density (g cm^{-3})	Deuterium uptake (cm^3 cm^{-3})	Selectivity (D_2/H_2) (1:1 Mixture)	Conditions
MFU-4l	8.30	0.56[1]	104.11	1.70[2]	40 K<br>10 mbar
MFU-4 (Zn, Cl)	1.24	1.48[3]	41.11	7.50[4]	60 K<br>10 mbar
MOF-5	11.15	0.61[5]	152.35	1.40[6]	70 K<br>10 mbar
py@COF-1	0.50	0.89[7]	9.97	9.70[8]	22 K<br>26 mbar
CC3	3.67	0.97[9]	79.74	1.70[10]	30 K<br>10 mbar
6FT-CC3	2.81	1.29[11]	81.20	2.20[10]	30 K<br>10 mbar
6ET-CC3	0.39	1.08[10]	9.43	3.90[10]	30 K<br>10 mbar
Cocryst 1	4.72	1.06[10]	112.07	8.00[10]	30 K<br>10 mbar
Ni_2Cl_2BBTA	1.65	~1.14[12]	42.13	4.50[13]	77 K<br>10 mbar
FJI-Y11	~8.00	1.20[14]	215.04	1.76[14]	77 K<br>1000 mbar
ECUT-8	~8.00	1.39[15]	249.09	1.40[15]	77 K<br>1000 mbar
Zeolite 5A	4.00	1.49[16]	133.50	2.70[17]	30 K<br>10 mbar
Zeolite (MS13X)	0.20	1.42[18]	6.36	3.05[19]	77 K<br>5 mbar
Y	2.44	1.43[20]	78.16	1.20[21]	77 K<br>175 mbar
SBA-15	1.74	<1.20[22]	46.77	1.25[21]	
10X	2.98	1.39[18]	92.78	1.17[21]	

Na-CHA	0.68	1.78[23]	27.11	0.96[24]	77 K 100 mbar
K-CHA	0.96	1.82[23]	39.13	0.98[24]	
Ca-CHA	4.70	1.78[23]	187.40	0.97[24]	
K-LTA	0.54	1.63[25]	19.72	1.08[24]	
NaX	0.40	1.42[18]	12.72	3.30[26]	77 K 2–5 mbar
LiX	0.40	1.29[18]	11.56	4.40[26]	
MgX	0.60	1.35[18]	18.14	5.80[26]	
MnX	0.80	1.46[18]	26.16	4.80[26]	
CaX	1.10	1.39[18]	34.25	3.60[26]	
KX	~0.60	1.57[18]	21.10	2.60[26]	
BaX	~1.50	1.84[18]	61.82	2.50[26]	
NaY	0.10	1.39[27]	3.11	2.70[26]	
DAY	0.40	1.33[27]	11.92	2.00[26]	
MFI-Si	0.90	1.76[28]	35.48	1.30[26]	
CHA-Si	0.50	1.54[23]	17.25	1.60[26]	
USTC-700	1.34	2.59	77.74	9.50	30 K 10 mbar

Supplementary Table 5 | The comparison of the costs (in GBP) to prepare one gram of each porous material for the KQS application. The lowest prices of major chemicals used for the syntheses that could be offered in China by commercial suppliers were quoted and calculated.

Compound	Raw materials	Cost (£/g raw materials)	Raw materials consumption (g/g sorbent)	Total cost (£/g sorbent)
MFU-4l[29]	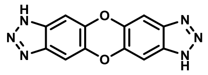 (Adamas)	182.68	0.81	148.06
	$ZnCl_2$ (Energy Chemical)	0.01	8.28	
MFU-4 (Zn, Cl)[3]	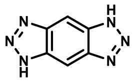 (Shaoyuan Co. Ltd.)	272.58	0.65	177.20
	$ZnCl_2$ (Energy Chemical)	0.01	2.19	
MOF-5[30]	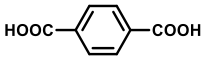 (Adamas)	0.01	0.89	0.26
	$Zn(NO_3)_2 \cdot 6H_2O$ (Aladdin)	0.05	4.86	
Ni_2Cl_2BBTA[13]	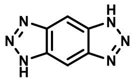 (Shaoyuan Co. Ltd.)	272.58	0.46	125.42
	$NiCl_2 \cdot 6H_2O$ (3A materials)	0.03	1.37	
FJI-Y11[14]	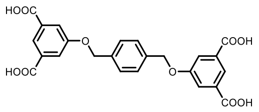 (Bide pharm)	135.92	1.59	216.44
	$Cu(NO_3)_2 \cdot 3H_2O$ (RHAWN)	0.13	2.47	
ECUT-8[15]	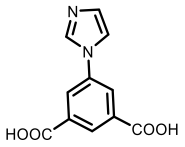 (Bide pharm)	111.14	0.81	90.82
	$Co(NO_3)_2 \cdot 6H_2O$	0.04	0.54	

	(kaiwei chemical)			
	$Th(NO_3)_2 \cdot 6H_2O$ (Macklin)	1.44	0.54	
COF-1[7]	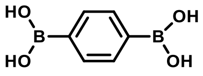 (Meryer)	0.98	1.47	1.44
CC3[9]	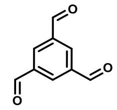 (Meryer)	25.94	3.24	85.58
	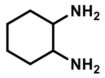 (kaiwei chemical)	0.45	3.4	
6ET-RCC3[10]	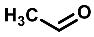 (Aladdin)	0.26	0.42	92.91
	RCC3*	88.38	1.05	
Cocryst 1[10]	CC3	85.58	0.47	90.39
	6ET-RCC3	92.91	0.54	
Ca-CHA[24]	Ultrastable zeolite Y (Aladdin)	0.02	0.88	0.03
	KOH (RHAWN)	0.01	0.76	
	$Ca(OH)_2$ (Macklin)	0.01	0.32	
MgX[26]	Zeolite NaX (kaiwei chemical)	0.01	1.06	6.56
	$Mg(NO_3)_2$ (Thermofisher scientific)	4.17	1.57	
USTC-700	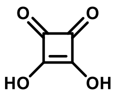 (Meryer)	0.73	0.93	0.72
	$TiOSO_4$ (Energy Chemical)	0.02	2.61	

*Synthesized compounds (not directly commercially available). Raw materials to prepare those linkers are summarized below.

RCC3[11]	CC3-R	85.58	1.03	88.38
	$NaBH_4$ (Energy Chemical)	0.21	1.11	

Supplementary Table 6 | Comparison of the space-time yields of various porous materials.

Compound	Space-time yield ($g\ m^{-3}\ day^{-1}$)
MFU-4l[29]	1650
MFU-4 (Zn, Cl)[3]	5170
MOF-5[30]	17760
Ni_2Cl_2BBTA[13]	1503
FJI-Y11[14]	576
ECUT-8[15]	1135
COF-1[7]	5670
CC3[9]	2083
6ET-RCC3[10]	841
Cocryst 1[10]	640
Ca-CHA[24]	11984
MgX[26]	15136
USTC-700	12240

Supplementary Table 7 | Unit cell parameters of the USTC-700 powder samples indexed from the capillary PXRD data collected at room temperature and 125 K.

	**Room temperature**	**125 K**
***a* (Å)**	3.700973	3.698424
***b* (Å)**	7.199039	7.196644
***c* (Å)**	6.429969	6.424316
***β* (°)**	106.64491	106.63935

Supplementary Table 8 | Summary of TDS measurement data on USTC-700 samples after chemical treatments.

Sample	Total Uptake (mmol g^{-1})	Selectivity
**Pristine**	1.07	7.3
**HNO_3-treated**	1.01	7.5
**Boiling Water-treated**	1.10	7.5

Supplementary Notes

Supplementary Note 1 | Methods

Materials: Squaric acid (Energy Chemical, 98%), $TiO(acac)_2$ (Macklin, 98%), $TiOSO_4·xH_2SO_4·xH_2O$ (Macklin, 93%), $Ti(OBu)_4$ (Acros, 98%+), $Ti(iPrO)_4$ (Alfa Aesar, 97%+), hydrochloric acid (Sinopharm, 37%), sulfuric acid (Sinopharm, 98%), phosphoric acid (Sinopharm, 85%), nitric acid (Sinopharm, 65%) were purchased from commercial suppliers and used as received without further purification.

Instruments: The powder X-ray diffraction data (PXRD) for general characterization and comparison were collected on a Rigaku SmartLab Diffractometer with Cu Kα radiation (λ = 1.54178 Å). The PXRD data to determine the zero thermal expansion of the USTC-700 structure were collected on an Oxford Cryosystems PheniX Diffractometer with Cu-Kα_1 radiation (λ = 1.54059 Å) at room temperature and 125 K separately. Thermogravimetric measurements were carried out with a Netzsch TG 209 F1 Libra thermal analyzer under air atmosphere, with a heating rate of 5 °C/min. Carbon dioxide sorption data were collected on a Micromeritics 3Flex analyzer at 273 K, as-made sample was activated under dynamic vacuum at 180 °C overnight prior to data collection. cRED data of USTC-700 was collected on JEOL 2100 Plus using ASI Cheetah120 detector and its crystallographic structure was further refined against high-quality data collected on the STOE STADI P ESSENTIAL X-ray diffractometer equipped with a Mythen II detector in the Debye-Scherrer mode with pure Cu Ka_1 radiation (λ: 1.5406 Å) (capillary: 0.2 mm, angle range: 5-120°, step size: 0.015°, total counting time: 78 h, room temperature).

Scale-up synthesis of USTC-700: To a 1 L round bottom flask, squaric acid (5.7 g, 50 mmol) and diluted HCl (3 M, 500 mL) were added and stirred at RT.

Titanium precursor (50 mmol) was added while stirring. The reaction mixture was refluxed at 120 °C for 24 h. After cooling to RT, the crude product of USTC-700 was collected by filtration, washed with EtOH and air dry.

Chemical stability: Batches of pristine sample of USTC-700 (250 mg) were refluxed in water (20 mL) and concentrated HCl (12 M, 20 mL) separately for three days in 50 mL round bottom flasks. Pristine sample of USTC-700 (250 mg) was soaked in aqueous solution (20 mL) for three days at room temperature in the cases of concentrated HNO_3 (15 M), concentrated H_2SO_4 (18 M), concentrated H_3PO_4 (18 M), aqua regia, basic buffer solution with a pH value of 12. Except for the samples after refluxed in water, all the other samples after chemical treatments were washed with plenty of water before filtration was applied to collect the products.

Mechanical stability: The effect of pressure applied on USTC-700 sample was studied. An increasing uniaxial compression, from 0 to 24 tonne cm^{-2}, was applied on 80 mg of sample using a hydraulic press and the sample pellet was grounded in a mortar before collecting the PXRD patterns. By using the full-width at half-maximum (FWHM) of the first PXRD peak collected on the pristine USTC-700 sample as the standard, the broadening of the first diffraction peaks (change of peak FWHM) of samples subjected to different pressures were normalized, analyzed and compared.

Supplementary Note 2 | Data collection and structure determination of USTC-700

cRED data of USTC-700 was collected on JEOL 2100 Plus equipped with ASI Cheetah120 detector. A high-quality 3D ED dataset (resolution: 0.9 Å; completeness: 85.1 %; 742 ED frames ranging from -53.4° to 64.3°) within three minutes using the cRED technique as displayed in Supplementary Table 1 and 2. The 3D reconstructed reciprocal lattice shown in Supplementary Figure 2a was processed by REDp software.[31] The reciprocal planes cut from 3D reconstructed reciprocal space are displayed in Supplementary Figure 2b-d. *hkl* intensities were extracted by employing XDS software.[32] Direct methods implemented in SHELXT were applied to solve its initial structure with the space group of *P*2/m (No. 10). The initial structural model of USTC-700 was further refined against high-quality PXRD data and final refinement results are shown in Supplementary Figure 2e and Supplementary Table 3.

Supplementary Note 3 | Hydrogen isotope adsorption/separation measurements

Hydrogen isotope adsorption: A fully automated Sierverts apparatus iQ2 (Quantachrome Instruments) was used to perform the adsorption experiments. The calibration cell is an empty analysis carried out at the same temperature and pressure range than each experiment; corrections related the sample volume and the non-linearity of the adsorbate are made. Around 20 mg of sample were activated at 393 K under vacuum for 5 hours in order to remove any solvent molecules. A coupled cryocooler based on the Gifford-McMahon cycle was used to control the sample temperature. The cooling system allows to measure temperatures from 20 to 300 K with an estimated error of < 0.05 K.

Calibration of the mass spectrometer signal: Calibration of the mass spectrometer signal. A solid piece of a diluted Pd alloy $Pd_{95}Ce_5$ (~0.5 g) was used for calibration. Before the calibration, the oxide layer of the alloy was removed by etching with aqua regia. Then the alloy was heated up to 600 K under high vacuum to remove any hydrogen that might be absorbed during the etching procedure. Afterwards, it was exposed to 40 mbar pure H_2 or pure D_2 for 1.5–2.5 h at 350 K after the mass had been collected. As H and D were bound preferentially to the Cerium atoms at low exposure pressures, the alloy could be handled under ambient conditions for a short time. The alloy was weighed after being cooled down to room temperature. The mass difference between unloaded state and loaded state was equal to the mass uptake of hydrogen or deuterium, respectively. After weighing, the alloy was loaded in the chamber again, and then a 0.1 $K \cdot s^{-1}$ heating ramp (RT to 600 K) was applied for a subsequent desorption spectrum. The obtained mass of gas is directly corresponded to the area under the desorption peak.

Supplementary Note 4 | Simulation and theoretical calculation

Geometry Optimization: DFT geometry optimization was performed with the Vienna Ab initio Simulation Package (VASP) employing plane-wave basis sets and projector augmented wave (PAW) potentials.[33,34] The general gradient approximation (GGA) to the exchange-correlation functional according to Perdew-Burke-Ernzerhof (PBE)[35] was used in combination with Grimme's empirical dispersion correction with Becke−Johnson damping (DFT-D3/BJ).[36,37] The atomic positions and lattice parameters of the frameworks were optimized with forces converged to 0.01 eV/Å for all atoms. A cutoff in the plane wave basis of 600 eV and a k-space resolution of ~0.1/Å in the reciprocal space was used.

Pore size distribution (PSD): Pore size distribution (PSD) of the optimized USTC-700 geometry was calculated with zeo++ code.[38]

Free energy profile: Free energy profiles of the H_2 and D_2 molecules along the 1-D channel of the USTC-700 MOF were calculated by Monte Carlo simulations in the canonical (NVT) ensemble applying the Widom's test particle insertion method using RASPA code.[39] During the course of the Monte Carlo simulation, a H_2 or D_2 molecule was attempted to insert into framework as many as 5×10^6 random positions to calculate energy required for those insertions. These energy values were then mapped into reaction coordinates along the three crystallographic axes. For this simulation we considered a simulation box made of 12 × 6 × 7 conventional unit cells and atoms were maintained at their initial positions. The interactions between the guest molecules (H_2 or D_2) and the MOF structure were described by a combination of site-to-site Lennard-Jones (LJ) potentials. Furthermore, van der Waals interactions were truncated at a cutoff distance of 12 Å. The Lennard-Jones cross-interaction parameters were calculated by means of the Lorentz-Berthelot mixing rules. All atoms of the MOF framework were described by single LJ sites with parameters taken from the

universal force field (UFF).[40] The single site LJ potential (σ = 2.958 Å, ϵ = 36.7 K) proposed by Michels et al.[41] was used to describe the H_2 (or D_2) molecule. The so-called Feynman–Hibbs (FH) semi-classical effective potentials[42] were used to account for the temperature-dependent nuclear quantum effects in this force field based simulations.

Path integral molecular dynamics (PIMD) simulations: Path integral molecular dynamics (PIMD) calculations have been performed using the code i-pi,[43] and atomic interaction forces have been calculated using Quickstep module[44] of the CP2K program[45,46] which is based on Gaussian Plane Wave (GPW) formalism. We have employed the general gradient approximation (GGA) to the exchange-correlation functional according to Perdew-Burke-Ernzerhof (PBE)[35] in a combination of Grimme's DFTD3 semi-empirical dispersion corrections.[36,37] Triple-ζ plus valence polarized Gaussian-type basis sets (TZVP-MOLOPT) were considered for all atoms, except for the Ti centers, where short ranged double-ζ plus valence polarization functions (DZVP-MOLOPT) were employed.[47] The interactions between core electrons and valence shells of the atoms were described by the pseudo-potentials derived by Goedecker, Teter, and Hutter (GTH).[48-50] The auxiliary plane wave basis sets were truncated at 400 Ry. PIGLET thermostat algorithm[51] and the system has been simulated in the NVT ensemble. In PIMD formalism, the system is replicated and all the replica of each atom is called a path integral ring polymer. For a classical molecule, the ring polymer gyration radius is null, the more the quantum nuclear effects are significant, the larger will be the gyration radius.

Supplementary Note 5 | Space-time yield (STY) calculation

Space-time yield is calculated as STY = $M_{product}$ / (T×V), Where $M_{product}$ is the mass of the product (g), T is the reaction duration (day), V is the reaction solvent volume (m^3).

For synthetic compounds that directly prepared using commercially available chemicals, the STY calculation only considers parameters of one-step reaction. For instance, the STY calculation of the porous organic cage compound CC3[9] is detailed as following: it was stated in the literature that the synthesis of CC3 requires 6 mL of CH_2Cl_2, 48 hours of reaction time. And finally, 25 mg of product could be obtained in one reaction. Thus, the STY of the CC3 synthesis could be calculated as:

$$M_{product} = 0.025\ g$$

$$T = 48/24 = 2\ days$$

$$V = 6\times10^{-6}\ m^3$$

$$STY_{CC3} = 0.025/(2\times6\times10^{-6}) = 2083\ g\ m^{-3}\ day^{-1}$$

For synthetic compounds whose organic linkers are not directly commercially available but are synthesized via multiple organic reactions and transformations, the corresponding STY calculations include not only the hybrid materials syntheses, but also the duration and yields of linker preparations should be considered and combined into the overall products yields and reaction time needed. For example, the STY calculation of the 6ET-RCC3[10] synthesis is detailed as following: it is stated in the literature that the synthesis of 6ET-RCC3 requires 500 mg of the RCC3, 30 mL of MeOH, 2 hours of reaction time while 472 mg of product can be obtained in one reaction. The synthesis of the RCC3 requires 926 mg of CC3, a total of 52 mL of solvent and a total reaction time of 24 hours, yielding 900 mg of RCC3 in one reaction. Correspondingly, the STY

of the overall preparation processes of RCC3 could be calculated as:

$$M_{product} = 0.472\ g$$

$$T = (2+24+48)/24 = 3.083\ days;$$

$$V = [30+52/(0.9/0.5)+926/(0.9/0.5)/25\times6)]\ mL = 1.82\times10^{-4}\ m^3$$

$$STY_{6ET\text{-}RCC3} = 0.472/(3.083\times1.82\times10^{-4}) = 841\ g\ m^{-3}\ day^{-1}$$

References

1 Denysenko, D., Grzywa, M., Tonigold, M., Streppel, B., Krkljus, I., Hirscher, M., Mugnaioli, E., Kolb, U., Hanss, J. & Volkmer, D. Elucidating Gating Effects for Hydrogen Sorption in MFU-4-Type Triazolate-Based Metal-Organic Frameworks Featuring Different Pore Sizes. *Chem. Eur. J.* **17**, 1837-1848 (2011).

2 Savchenko, I., Mavrandonakis, A., Heine, T., Oh, H., Teufel, J. & Hirscher, M. Hydrogen isotope separation in metal-organic frameworks: Kinetic or chemical affinity quantum-sieving? *Microporous Mesoporous Mater.* **216**, 133-137 (2015).

3 Biswas, S., Grzywa, M., Nayek, H. P., Dehnen, S., Senkovska, I., Kaskel, S. & Volkmer, D. A cubic coordination framework constructed from benzobistriazolate ligands and zinc ions having selective gas sorption properties. *Dalton Trans*, 6487-6495 (2009).

4 Teufel, J., Oh, H., Hirscher, M., Wahiduzzaman, M., Zhechkov, L., Kuc, A., Heine, T., Denysenko, D. & Volkmer, D. MFU-4-a metal-organic framework for highly effective H_2/D_2 separation. *Adv. Mater.* **25**, 635-639 (2013).

5 Li, H., Eddaoudi, M., O'Keeffe, M. & Yaghi, O. M. Design and synthesis of an exceptionally stable and highly porous metal-organic framework. *Nature* **402**, 276-279 (1999).

6 Teufel, J. Experimental investigation of H_2D_2 isotope separation by cryoadsorption in metal-organic frameworks. Universität Stuttgart (2013).

7 Cote, A. P., Benin, A. I., Ockwig, N. W., O'Keeffe, M., Matzger, A. J. & Yaghi, O. M. Porous, crystalline, covalent organic frameworks. *Science* **310**, 1166-1170 (2005).

8 Oh, H., Kalidindi, S. B., Um, Y., Bureekaew, S., Schmid, R., Fischer, R. A. & Hirscher, M. A cryogenically flexible covalent organic framework for efficient hydrogen isotope separation by quantum sieving. *Angew. Chem. Int. Ed.* **52**, 13219-13222 (2013).

9 Tozawa, T., Jones, J. T., Swamy, S. I., Jiang, S., Adams, D. J., Shakespeare, S., Clowes, R., Bradshaw, D., Hasell, T., Chong, S. Y., Tang, C., Thompson, S., Parker, J., Trewin, A., Bacsa, J., Slawin, A. M., Steiner, A. & Cooper, A. I. Porous organic cages. *Nat. Mater.* **8**, 973-978 (2009).

10 Liu, M., Zhang, L., Little, M. A., Kapil, V., Ceriotti, M., Yang, S., Ding, L., Holden, D. L., Balderas-Xicohtencatl, R., He, D., Clowes, R., Chong, S. Y., Schutz, G., Chen, L., Hirscher, M. & Cooper, A. I. Barely porous organic cages for hydrogen isotope separation. *Science* **366**, 613-620 (2019).

11 Liu, M., Little, M. A., Jelfs, K. E., Jones, J. T., Schmidtmann, M., Chong, S. Y., Hasell, T. & Cooper, A. I. Acid- and base-stable porous organic cages: shape persistence and pH stability via post-synthetic "tying" of a flexible amine cage. *J. Am. Chem. Soc.* **136**, 7583-7586 (2014).

12 Liao, P.-Q., Chen, H., Zhou, D.-D., Liu, S.-Y., He, C.-T., Rui, Z., Ji, H., Zhang, J.-P. & Chen, X.-M. Monodentate hydroxide as a super strong yet reversible active site for CO_2 capture from high-humidity flue gas. *Energy Environ. Sci.* **8**, 1011-1016 (2015).

13 Li, X., Wang, X., Li, M., Luo, J., An, Y., Li, P., Song, J., Chen, C., Feng, X. & Wang, S. Highly selective adsorption of D_2 from hydrogen isotopes mixture in a robust metal bistriazolate framework with open metal sites. *Int. J. Hydrogen Energy* **45**, 21547-21554 (2020).

14 Si, Y., He, X., Jiang, J., Duan, Z., Wang, W. & Yuan, D. Highly effective H_2/D_2 separation in a stable Cu-based metal-organic framework. *Nano Res.* **14**, 518-525 (2021).

15 Yin, M., Krishna, R., Wang, W., Yuan, D., Fan, Y., Feng, X., Wang, L. & Luo, F. A Th_8Co_8 Nanocage-Based Metal Organic Framework with Extremely Narrow Window but Flexible Nature Enabling Dual-Sieving Effect for Both Isotope and Isomer Separation. *CCS Chem.* **4**, 1016-1027 (2022).

16 Adams, J. M. & Haselden, D. A. The structure of dehydrated zeolite 5A (Si/Al = 1.02) by neutron profile refinement. *J. Solid State Chem.* **51**, 83-90 (1984).

17 Xiong, R., Balderas Xicohténcatl, R., Zhang, L., Li, P., Yao, Y., Sang, G., Chen, C., Tang, T., Luo, D. & Hirscher, M. Thermodynamics, kinetics and selectivity of H_2 and D_2 on zeolite 5A below 77K. *Microporous Mesoporous Mater.* **264**, 22-27 (2018).

18 Olson, D. H. A reinvestigation of crystal structure of zeolite hydrated NaX. *J. Phys. Chem.* **74**, 2758 (1970).

19 Niimura, S., Fujimori, T., Minami, D., Hattori, Y., Abrams, L., Corbin, D., Hata, K. & Kaneko, K. Dynamic Quantum Molecular Sieving Separation of D_2 from H_2-D_2 Mixture with Nanoporous Materials. *J. Am. Chem. Soc.* **134**, 18483-18486 (2012).

20 Parise, J. B., Corbin, D. R., Abrams, L. & Cox, D. E. Structure of dealuminated Linde Y-zeolite: $Si_{139.7}Al_{52.3}O_{384}$ and $Si_{173.1}Al_{18.9}O_{384}$ presence of non-framework Al species. *Acta Crystallogr. Sect. C- Cryst. Struct. Commun.* **40**, 1493-1497 (1984).

21 Chu, X.-Z., Cheng, Z.-P., Xiang, X.-X., Xu, J.-M., Zhao, Y.-J., Zhang, W.-G., Lu, J.-S., Zhou, Y.-P., Zhou, L., Moon, D.-K. & Lee, C.-H. Separation dynamics of hydrogen isotope gas in mesoporous and microporous adsorbent beds at 77 K: SBA-15 and zeolites 5A, Y, 10X. *Int. J. Hydrogen Energy* **39**, 4437-4446 (2014).

22 Yahiaoui, O., Fitch, A. N., Hoffman, F., Froeba, M., Thomas, A. & Roeser, J. 3D Anionic Silicate Covalent Organic Framework with srs Topology. *J. Am. Chem. Soc.* **140**, 5330-5333 (2018).

23 Diaz-Cabanas, M. J., Barrett, P. A. & Camblor, M. A. Synthesis and structure of pure SiO_2 chabazite: the SiO_2 polymorph with the lowest framework density. *Chem. Commun.* **17**, 1881-1882 (1998).

24 Taguchi, A., Nakamori, T., Yoneyama, Y., Sugiyama, T., Tanaka, M., Kotoh, K., Tachibana, Y. & Suzuki, T. Hydrogen Isotope (H_2 and D_2) Sorption Study of CHA-Type Zeolites. *Fusion Sci. Technol.* **76**, 314-320 (2020).

25 Adams, J. M. & Haselden, D. A. The structure of dehydrated zeolite 3A (Si/Al = 1.01) by neutron profile refinement. *J. Solid State Chem.* **47**, 123-131 (1983).

26 Giraudet, M., Bezverkhyy, I., Weber, G., Dirand, C., Macaud, M. & Bellat, J.-P. D_2/H_2 adsorption selectivity on FAU zeolites at 77.4 K: Influence of Si/Al ratio and cationic composition. *Microporous Mesoporous Mater.* **270**, 211-219 (2018).

27 Hriljac, J. A., Eddy, M. M., Cheetham, A. K., Donohue, J. A. & Ray, G. J. Powder neutron-diffraction and Si-29 mas nmr-studies of siliceous zeolite-Y. *J. Solid State Chem.* **106**, 66-72 (1993).

28 Flanigen, E. M., Bennett, J. M., Grose, R. W., Cohen, J. P., Patton, R. L., Kirchner, R. M. & Smith, J. V. Silicalite, a new hydrophobic crystalline silica molecular-sieve. *Nature* **271**, 512-516 (1978).

29 Denysenko, D., Grzywa, M., Tonigold, M., Streppel, B., Krkljus, I., Hirscher, M.,

Mugnaioli, E., Kolb, U., Hanss, J. & Volkmer, D. Elucidating gating effects for hydrogen sorption in MFU-4-type triazolate-based metal-organic frameworks featuring different pore sizes. *Chemistry* **17**, 1837-1848 (2011).
30 Kaye, S. S., Dailly, A., Yaghi, O. M. & Long, J. R. Impact of preparation and handling on the hydrogen storage properties of Zn_4O(1,4-benzenedicarboxylate)$_3$ (MOF-5). *J. Am. Chem. Soc.* **129**, 14176-14177 (2007).
31 Wan, W., Sun, J., Su, J., Hovmoller, S. & Zou, X. Three-dimensional rotation electron diffraction: software RED for automated data collection and data processing. *J. Appl. Crystallogr.* **46**, 1863-1873 (2013).
32 Kabsch, W. Integration, scaling, space-group assignment and post-refinement. *Acta Crystallogr. Sect. D-Biol. Crystallogr.* **66**, 133-144 (2010).
33 Kresse, G. & Furthmuller, J. Efficient iterative schemes for ab initio total-energy calculations using a plane-wave basis set. *Phys. Rev. B* **54**, 11169-11186 (1996).
34 Kresse, G. & Joubert, D. From ultrasoft pseudopotentials to the projector augmented-wave method. *Phys. Rev. B* **59**, 1758-1775 (1999).
35 Perdew, J. P., Burke, K. & Ernzerhof, M. Generalized gradient approximation made simple. *Phys. Rev. Lett.* **77**, 3865-3868 (1996).
36 Grimme, S., Antony, J., Ehrlich, S. & Krieg, H. A consistent and accurate ab initio parametrization of density functional dispersion correction (DFT-D) for the 94 elements H-Pu. *J. Chem. Phys.* **132**, 154104 (2010).
37 Grimme, S. Accurate description of van der Waals complexes by density functional theory including empirical corrections. *J. Comput. Chem.* **25**, 1463-1473 (2004).
38 Willems, T. F., Rycroft, C., Kazi, M., Meza, J. C. & Haranczyk, M. Algorithms and tools for high-throughput geometry-based analysis of crystalline porous materials. *Microporous Mesoporous Mater.* **149**, 134-141 (2012).
39 Dubbeldam, D., Calero, S., Ellis, D. E. & Snurr, R. Q. RASPA: molecular simulation software for adsorption and diffusion in flexible nanoporous materials. *Mol. Simul.* **42**, 81-101 (2016).
40 Rappe, A. K., Casewit, C. J., Colwell, K. S., Goddard, W. A. & Skiff, W. M. UFF, a full periodic-table force-field for molecular mechanics and molecular-dynamics simulations. *J. Am. Chem. Soc.* **114**, 10024-10035 (1992).
41 Michels, A., Degraaff, W. & Tenseldam, C. A. Virial coefficients of hydrogen and deuterium at temperatures between -175 °C and +150 °C. Conclusions from the second virial coefficient with regards to the intermolecular potential. *Physica* **26**, 393-408 (1960).
42 Feynman, R. P. & Hibbs, A. R. Quantum mechanics and path integrals. (McGraw-Hill, New York, 1965).
43 Kapil, V., Rossi, M., Marsalek, O., Petraglia, R., Litman, Y., Spura, T., Cheng, B., Cuzzocrea, A., Meissner, R. H., Wilkins, D. M., Helfrecht, B. A., Juda, P., Bienvenue, S. P., Fang, W., Kessler, J., Poltavsky, I., Vandenbrande, S., Wieme, J., Corminboeuf, C., Kuehne, T. D., Manolopoulos, D. E., Markland, T. E., Richardson, J. O., Tkatchenko, A., Tribello, G. A., Van Speybroeck, V. & Ceriotti, M. i-PI 2.0: A universal force engine for advanced molecular simulations. *Comput. Phys. Commun.* **236**, 214-223 (2019).
44 VandeVondele, J., Krack, M., Mohamed, F., Parrinello, M., Chassaing, T. & Hutter, J.

QUICKSTEP: Fast and accurate density functional calculations using a mixed Gaussian and plane waves approach. *Comput. Phys. Commun.* **167**, 103-128 (2005).

45 Hutter, J., Iannuzzi, M., Schiffmann, F. & VandeVondele, J. CP2K: atomistic simulations of condensed matter systems. *Wiley Interdiscip. Rev.- Comput. Mol. Sci.* **4**, 15-25 (2014).

46 The CP2K developers group, <http://www.cp2k.org> (accessed Feb 10,2019).

47 VandeVondele, J. & Hutter, J. Gaussian basis sets for accurate calculations on molecular systems in gas and condensed phases. *J. Chem. Phys.* **127** (2007).

48 Goedecker, S., Teter, M. & Hutter, J. Separable dual-space Gaussian pseudopotentials. *Phys. Rev. B* **54**, 1703-1710 (1996).

49 Krack, M. Pseudopotentials for H to Kr optimized for gradient-corrected exchange-correlation functionals. *Theor. Chem. Acc.* **114**, 145-152 (2005).

50 Hartwigsen, C., Goedecker, S. & Hutter, J. Relativistic separable dual-space Gaussian pseudopotentials from H to Rn. *Phys. Rev. B* **58**, 3641-3662 (1998).

51 Ceriotti, M. & Manolopoulos, D. E. Efficient First-Principles Calculation of the Quantum Kinetic Energy and Momentum Distribution of Nuclei. *Phys. Rev. Lett.* **109**, 100604 (2012).
